# Combining transcranial direct current stimulation and computerized cognitive training for post-stroke cognitive recovery: Study protocol for a randomized controlled trial with optimized temporal intervention sequences

**DOI:** 10.1371/journal.pone.0342761

**Published:** 2026-03-04

**Authors:** Xinling Wei, Jianwei Xia, Rui Luo, Peng Hu, Yuanyuan Wu, Jia Liu, Luping Song, Naifu Jiang, Minghong Sui

**Affiliations:** 1 Rehabilitation College, Gannan Medical University, Jiangxi, China; 2 School of Special Education and Rehabilitation, Binzhou Medical University, Yantai, China; 3 Department of Medicine, Shenzhen University, Shenzhen, China; 4 Clinical College, Youjiang Medical University For Nationalities, Baise, China; 5 Rehabilitation Medicine Department, Shenzhen Nanshan People’s Hospital(Shenzhen Sixth People’s Hospital), Shenzhen, China; 6 Guangdong-Hong Kong-Macao Joint Laboratory of Human-Machine Intelligence-Synergy Systems, Shenzhen Institutes of Advanced Technology Chinese Academy of Sciences, Shenzhen, China; Iran University of Medical Sciences, IRAN, ISLAMIC REPUBLIC OF

## Abstract

**Background:**

Post-stroke cognitive impairment is a common and severe complication of stroke, significantly affecting patients’ daily life and rehabilitation. Although transcranial direct current stimulation combined with computerized cognitive training has shown potential for improvement, current studies still have key limitations. Most have used only simultaneous intervention protocols, without systematically comparing sequential combination strategies, making it difficult to determine the optimal clinical approach.

**Objectives:**

The core objective of this study will be to address the existing research gap by comparing sequential intervention strategies and to resolve the key issue of identifying the optimal clinical approach.

**Methods:**

This study will use a single-center, double-blind, randomized controlled trial design. A total of 60 patients with post-stroke cognitive impairment will be randomly assigned in a 1:1:1:1 ratio to four groups: three intervention groups receiving computerized cognitive training combined with transcranial direct current stimulation at different timings (pre-training, concurrent, or post-training), and one sham control group. All interventions will be administered five times a week for two consecutive weeks. Follow-up assessments will be conducted at 4 and 12 weeks post-intervention.

**Discussion:**

This trial will systematically evaluate the optimal timing strategies for combining tDCS and CCT in PSCI. The hypothesized mechanisms are: (1) synchronous stimulation enhancing cognitive processing efficiency through immediate synaptic changes, (2) pre-training stimulation promoting plasticity via long-term potentiation-like modifications, and (3) post-training stimulation consolidating training effects by modulating neuronal synchrony. High-sensitivity cerebral oxygenation parameters will be used to assess microcirculatory improvements, linking physiological changes to cognitive recovery. However, ceiling effects and test biases will potentially limit the accuracy of long-term results, while sample size and lack of stratification based on brain lesion characteristics may introduce heterogeneity. Future studies will need to incorporate multimodal neuroimaging for stratified analysis and should develop personalized interventions based on lesion severity, disease stage, and timing to address therapeutic challenges and advance precision rehabilitation.

**Trial registration:** ChiCTR2500102565.[Chinese Clinical Trial Registry (ChiCTR), https://www.chictr.org.cn/] [Registered on May 16, 2025]

## Introduction

Stroke, the second leading cause of death worldwide, significantly affects patients and their families due to its high incidence and severe long-term consequences [1].Survivors often experience lingering motor, sensory, cognitive, and bowel/bladder dysfunctions, which reduce their quality of life, impose a significant economic burden on society, and cause emotional strain on families [2,3].International studies show that the incidence of post-stroke cognitive impairment (PSCI) in the Chinese population can reach 78.7% within 3 months post-stroke [4].Cognitive impairments, including severe declines in memory, attention, and executive function, make it difficult for patients to perform daily tasks. The distress caused by these impairments can sometimes outweigh that of physical disabilities, complicating rehabilitation and hindering overall recovery. Given the multidimensional impact of cognitive dysfunction on rehabilitation, cognitive recovery should be prioritized as highly as physical restoration [5,6].

The James Lind Alliance, a UK-based partnership for setting research priorities, has identified PSCI as a top priority in stroke research [7].The 2021 joint guidelines from the European Stroke Organisation and European Academy of Neurology highlight the ongoing therapeutic limitations of cognitive training for PSCI. To overcome this challenge, the guidelines emphasize the need for prioritizing randomized controlled trials in future research [8].Computerized cognitive training (CCT) is a software-based intervention delivered through digital platforms, known for its engaging interface, portability, and real-time progress tracking. This approach has gained popularity in clinical practice and shown significant therapeutic efficacy in cognitive impairment rehabilitation [9].Its potential benefits may arise from the interaction between cognitive-psychological changes induced by CCT and dopamine neurotransmitter release [10].However, its use as a standalone treatment for PSCI patients shows limited clinical efficacy in certain populations [11].This limitation arises from CCT’s unimodal design (e.g., visual tasks), which restricts the activation of multimodal neural circuits and hinders neuroplasticity. Additionally, standalone CCT provides short-term cognitive benefits but struggles to generalize domain-specific improvements to overall functional gains [12].

To overcome these limitations, combining therapy with non-invasive brain stimulation has become a key research focus. Transcranial Direct Current Stimulation (tDCS) is especially prioritized due to its safety profile—it does not induce action potentials, which significantly reduces the risk of seizures and ensures treatment safety [13].Additionally, tDCS effectively modulates cortical excitability, addressing CCT’s limitations by regulating neuroplasticity in a targeted manner, thereby creating a ‘neuroactivation-cognitive enhancement’ feedback loop [14,15].tDCS also exhibits significant long-lasting aftereffects, with 20–30 minutes of stimulation maintaining neural network modulation for up to 90 minutes post-treatment, thereby providing a neurobiological basis for sustained cognitive enhancement [16].However, most devices lack real-time monitoring and adaptive parameter adjustments (e.g., stimulus intensity), which limits their regulatory efficacy beyond simple intervention-based stimulation.

Current evidence suggests that combined therapy typically enhances efficacy compared to monotherapy, though study conclusions remain inconsistent.Chen et al. [17]observed significantly improved vascular vasomotor function in the combined therapy group via transcranial Doppler ultrasonography, linked to optimized hippocampal activation patterns and a bidirectional beneficial cycle between vascular and cognitive functions. Notably, Kolskar et al. [18] reported no additional benefits of tDCS-CCT combinations over CCT alone in high-reliability fMRI-based brain activation analyses.Meanwhile, studies confirm significant differences in clinical outcomes between combined strategies.Naro et al. [19]found both concurrent tDCS-RAGT and sequential RAGT-then-tDCS interventions in stroke patients significantly improved balance (p < 0.001) and moderately enhanced gait endurance (p = 0.04), with the sequential approach showing particularly sustained effects—contradicting Leon’s null findings [20].Current investigations into tDCS-CCT combinations highlight potential cognitive benefits, yet their efficacy remains inconsistent [21].A meta-analysis confirmed significant improvements in working memory with CCT in stroke patients, but there were insufficient tDCS studies for analysis, and meta-regression identified no significant moderators.Given that tDCS and CCT exhibit complementary mechanisms of action and that controversy exists regarding the efficacy of combined tDCS at different time sequences., we will propose combining these interventions to address cognitive impairments in stroke patients [22].

Given the significant gap in research on combined tDCS-CCT intervention strategies, particularly the lack of systematic exploration into the differential effects between simultaneous and sequential intervention modes, this study will investigate the cognitive intervention effects of tDCS in stroke patients undergoing CCT. The primary objective will be to compare efficacy differences across various temporal combinations of tDCS-CCT therapies to identify the optimal synergistic protocol. Rooted in the theoretical framework linking tDCS-mediated cerebral hemodynamic modulation to cognitive enhancement, the study will specifically examine the association between prefrontal cortical oxygen metabolism and cognitive improvement. Functional Near-Infrared Spectroscopy (fNIRS) will be used to dynamically monitor pre- and post-treatment hemodynamic parameters in the prefrontal cortex, aiming to elucidate the neurophysiological mechanisms underlying cognitive recovery through the lens of neurovascular coupling.

## Methods and design

The study protocol was approved by the Scientific Research Ethics Committee of Nanshan District People’s Hospital, Shenzhen (Approval Number: Scientific Ethics Review ky-2024 103101, Approval Date: March 5, 2025), and registered with the Chinese Clinical Trial Registry (ChiCTR) prior to the initiation of the study (Registration Number: ChiCTR2500102565, Registration Date: May 16, 2025)https://www.chictr.org.cn/. All study activities will be conducted in accordance with the requirements of the ethics committee, and the validity of the study protocol will cover the entire duration of the research (until March 5, 2026).

This study will be in the participant recruitment phase, which is expected to be completed between June 2025 and July 2026. At the time of submission, data collection has not yet commenced. As a study protocol, this manuscript will aim to describe the planned study prior to the generation of any results.

### Study design

**Fig 1:**
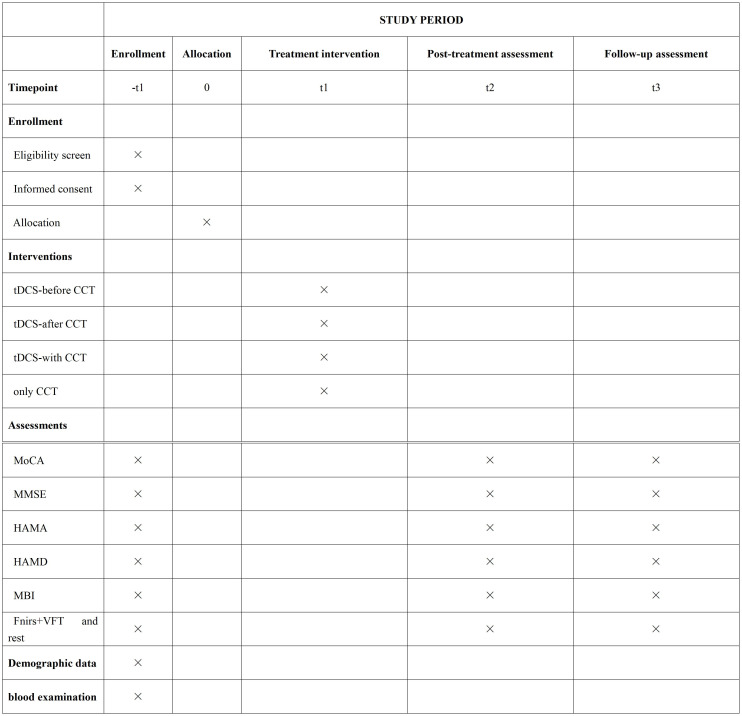
Trial schedule of enrollment, intervention, and assessments. Trial schedule of enrollment, intervention, and assessments.MoCA,Montreal Cognitive Assessment; MMSE,Mini-Mental State Examination; HAMA,Hamilton Anxiety Scale; HAMD,Hamilton Depression Rating Scale;MBI,Modified Barthel Index; tDCS,Transcranial Direct Current Stimulation; CCT,Computerized Cognitive Training; fNIRS,Functional Near-Infrared Spectroscopy; VFT,Verbal Fluency Test.

**Fig 2:**
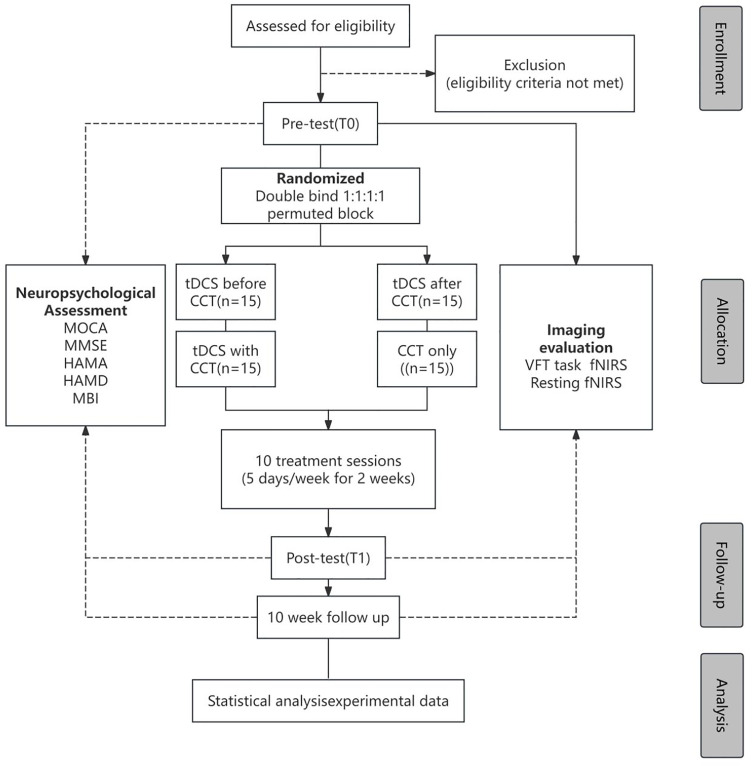
The CONSORT (Consolidated Standards of Reporting Trials) flowchart for clinical trials.

This is a single-center, double-blind, randomized controlled exploratory trial. Sixty patients will be randomly assigned to one of four groups (n = 15 per group). The protocol will be reported following Standard Protocol Items: Recommendations for Interventional Trials (SPIRIT) schedule statement(S1 File).Participant enrollment, randomization, assessment, and follow-up schedules are summarized in **Fig 1**.For a detailed visualization of the study workflow, **Fig 2**, which outlines the sequential steps from participant recruitment to final follow-up.

### Study setting and recruitment

Patient recruitment will be conducted at the Department of Rehabilitation Medicine, Shenzhen Nanshan People’s Hospital, from June 2025 to July 2026, using a dual approach to optimize eligibility assessment and engagement. Outpatient recruitment will utilize posters in the rehabilitation clinic, enabling interested patients to notify therapists for referral to the principal investigator for preliminary screening. Concurrently, resident physicians in the Neurology and Rehabilitation Medicine Departments will systematically screen inpatients against predefined criteria and refer eligible candidates to the research team for formal enrollment. All referring clinicians will undergo standardized protocol training to ensure consistency, and written informed consent will be obtained prior to participant inclusion. Eligibility criteria are detailed below.

### Participants

#### Inclusion criteria.

(a) Diagnosed with stroke (intracerebral hemorrhage or cerebral infarction) confirmed by head CT/MRI, per the 1995 Fourth National Cerebrovascular Disease Conference criteria;
(b) Alert, hemodynamically stable, and capable of completing cognitive assessments;
(c) First-time stroke within 3 months post-onset;
(d) MoCA score between 10–26 (inclusive);
(e) Voluntary informed consent.

#### Exclusion criteria:.

(a) History of other neurological disorders (e.g., Parkinson’s disease, tumors, hydrocephalus, or trauma);
(b) Cognitive impairment attributable to non-stroke conditions;
(c) Significant auditory/visual deficits, aphasia, or uncooperativeness;
(d) Pre-stroke cognitive decline.

#### Removal criteria:.

(a) Poor compliance or non-adherence to the protocol;
(b) Withdrawal of consent;
(c) Incomplete data preventing efficacy evaluation.

### Randomization

In this study, we’ll use blocked randomization and IBM SPSS Statistics 26.0 (International Business Machines Corporation, Armonk, NY, USA) for a 1:1:1:1 balanced allocation across four groups (pre-, concurrent-, post-training stimulation, and sham control).First, set a fixed random seed (based on the date for reproducibility). With N = 60 and balance needs, define block length as 12 (3 per group) and generate sequences for 5 blocks.Then, use rv.random(1,4) for random integers and sort randomly within blocks.Finally, match sequences with subject numbers (1–60) via match files and sort cases, assigning groups by block and order to control time trend interference.

### Allocation concealment

To maintain allocation concealment, a pre-generated randomization sequence will be embedded in sequentially numbered, opaque, sealed envelopes. These envelopes will be securely stored and accessed exclusively by designated therapists responsible for stratified randomization of eligible patients into study arms.

### Blinding

Throughout the trial, participants, outcome assessors, and data analysts will remain blinded to group assignments until final database lock.Unblinding will be permitted solely under emergency circumstances (e.g., serious adverse events). No interim unblinding procedures are required, as all data analyses will be conducted following trial completion.

### Sample size estimation

Prior to study initiation, we will perform sample size estimation using G*Power 3.1.9.7 (Heinrich Heine-Universität Düsseldorf, Düsseldorf, Germany). The primary outcome will be the MoCA score, and the effect-size parameter will be set at Cohen’s f = 0.708, as reported in a peer-reviewed publication [17]. The statistical model will be a linear mixed model testing the group × time interaction. The following settings will be used: power = 0.95, α = 0.05, four groups, three repeated measurements (baseline, post-treatment, and follow-up), and a within-subject correlation of 0.30 (reflecting an expected increase in between-subject variability at follow-up). The software will indicate that a total of 48 participants will be required. Anticipating a 20% dropout rate, we will divide 48 by 0.8 and round up to the nearest multiple of four, resulting in a planned recruitment of 60 participants (15 per group).

### Interventions

All participants will receive five sessions per week for two weeks, totaling 10 sessions. Adverse events will be monitored during each session, with researchers administering tDCS stimulation according to Brunoni et al.‘s protocol [23] to assess patient discomfort, pain, and any causality related to tDCS. Four treatment groups will be established:

1. Group A (tDCS-before-CCT): Patients will receive 20 minutes of tDCS, followed by 30 minutes of CCT after a 4-hour interval.
2. Group B (tDCS-after-CCT): Patients will complete 30 minutes of CCT first, then will receive 20 minutes of tDCS after a 4-hour interval.
3. Group C (tDCS-with-CCT): tDCS will be administered concurrently during the last 20 minutes of the 30-minute CCT session.
4. Group D (CCT-only/Sham): Patients will undergo CCT with simultaneous placebo tDCS (sham stimulation).

Prior to the treatment intervention, patients will undergo a baseline assessment to determine eligibility. Enrollment will be based on the Montreal Cognitive Assessment (MoCA) [24]. Assessors will be blinded to group allocation. Post-treatment assessments will be conducted immediately after treatment completion, with follow-up assessments scheduled at 4 and 12 weeks post-treatment.The use of any non-invasive brain stimulation protocols other than those explicitly specified in this study (e.g., transcranial magnetic stimulation [TMS]) is strictly prohibited throughout the research period.

#### tDCS.

**Fig 3:**
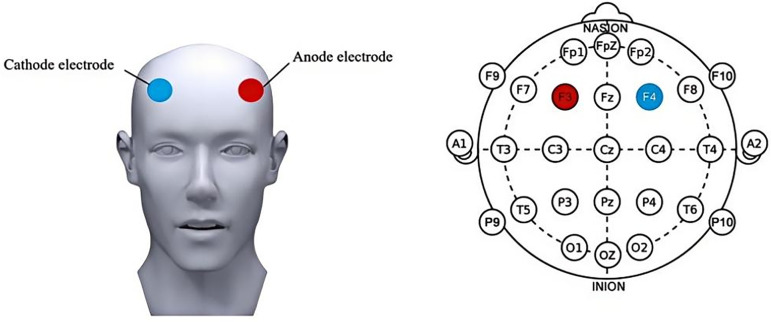
Schematic diagram of the stimulation area for tDCS.

The MBM-1 intelligent electrostimulator (Jiangxi Huaheng Jingxing Medical Technology Co., Ltd.) will be used, with 4.5 × 5.5 cm isotonic gelatin sponge electrodes soaked in saline (0.9% NaCl). Stimulation will target the dorsolateral prefrontal cortex (DLPFC) based on the EEG 10–20 system, with F3 as the anode and F4 as the cathode (**Fig 3**). Parameters: 1 mA current intensity (selected for safety and demonstrated behavioral effects) [25].Each session will last 20 minutes, with 5 sessions per week for 2 weeks.Ramp-Up/Ramp-Down: The current will gradually increase to 1 mA over the first 30 seconds and decrease to 0 mA during the final 30 seconds of each session. For sham stimulation, the device will activate for 30 seconds before automatically shutting down.

#### CCT.

The PASS-CATS 1.0 software will simulate cognitive tasks targeting learning/memory and executive inhibition.Learning/Memory Module: Object relocation, pattern recognition, novel character identification, numerical matching, mental arithmetic recall, and facial memory tasks.Executive Inhibition Module: Error detection, selective attention, symbol-number matching, linguistic inference, pattern reasoning, and numerical sequence tasks.Training sessions will last 30 minutes, administered 5 times per week for 2 weeks. The software will automatically record performance data, which will be scored by a blinded therapist.

### Outcome measures

#### Primary outcomes.

**Fig 4:**
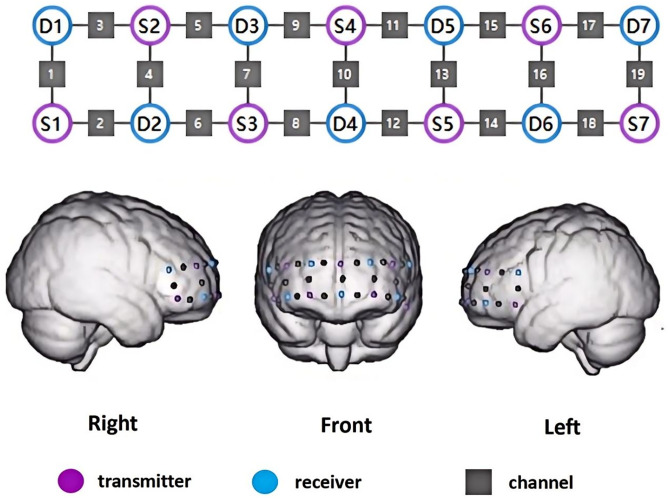
fNlRS Light Source Arrangement Diagram.

1. MoCA: will be administered at baseline, 4 weeks, and 12 weeks, with a focus on detecting mild cognitive improvements. The cutoff scores for cognitive impairment will be determined based on educational attainment (e.g., ≤ 13 points for primary education, ≤ 19 points for secondary education, and ≤24 points for tertiary education, as per the MoCA-Chinese version criteria).
2. Prefrontal Cortex (PFC) Activation (fNIRS): Task-related oxygenated hemoglobin (HbO) signals will be measured using the NirSmartⅡ-3000D system (Huichuang Medical, Danyang), with wavelengths of 730 nm/850 nm and a sampling rate of 11 Hz. The channel configuration will consist of 22 channels using 8 emitters and 8 detectors (3 cm separation), aligned with the EEG 10–20 system (**Fig 4**).The protocol includes 8 minutes of resting-state data (eyes closed, seated comfortably in a quiet room). The Verbal Fluency Task (VFT) will involve 30 seconds of pre-task rest, 60 seconds of task phase (generate words starting with |d|, |g|, or |h|; 20 seconds per phoneme), and 75 seconds of post-task rest. The outcomes will include PFC activation intensity, resting-state functional connectivity (left/right PFC), and word-generation accuracy.

#### Secondary outcomes.

1. Mini-Mental State Examination (MMSE): evaluates cognitive function (scored 0–30) and will be administered at baseline, 4 weeks, and 12 weeks. A score of ≥23 indicates normal cognition.
2. Barthel Index (BI): evaluates activities of daily living (ADL) with dependency levels classified as follows: independent (100 points), mild dependency (61–99 points), moderate dependency (41–60 points), and severe dependency (≤40 points).
3. Hamilton Anxiety Scale (HAMA):Scores ≥25 = severe anxiety; 21–24 = moderate anxiety; 14–20 = mild anxiety; 7–13 = borderline anxiety; < 7 = no anxiety.
4. Hamilton Depression Scale (HAMD): Total scores: 0–54 (higher = worse). ≥ 25 = severe depression; 18–24 = moderate depression; 7–17 = mild depression.
5. Follow-Up: MoCA and MMSE will be repeated at 4 and 12 weeks post-treatment to assess sustained cognitive improvements.

### Safety and adverse event monitoring

Participant safety will be monitored continuously throughout the 1-week intervention period and the 2-month follow-up phase. All adverse events (AEs) and serious adverse events (SAEs) – whether reported by participants or observed by study investigators – will be systematically documented in accordance with established clinical research protocols.To monitor and manage adverse effects, patients will report sensations of itching, tingling, burning, headache, or other discomforts using a 4-point scale (1 = absent, 2 = mild, 3 = moderate, 4 = severe). The causal relationship between symptoms and tDCS stimulation will be assessed on a 5-point scale (1 = unrelated, 5 = strongly related).In the event of adverse events (AEs) or serious adverse events (SAEs), investigators shall provide immediate appropriate medical intervention and conduct follow-up assessments until clinical resolution or stabilization is confirmed. SAE data will be formally reported during regular meetings of the Data Safety Monitoring Board (DSMB) in compliance with institutional safety protocols.

### Data management

Standardized eCRFs will capture baseline demographics, clinical parameters (stroke subtype, lesion site, cognitive status), labs, and treatment history from EMRs post-enrollment. AEs/SAEs will be tracked throughout treatment, with 3-month cognitive follow-up (MoCA, MMSE).Primary source data for this study will be maintained through secure archiving of paper CRFs containing participant information in locked cabinets within controlled-access facilities, while two trained data clerks under independent QA (JL and YYW) supervision will enter all CRF data into the EDC system, which automatically generates immutable audit trails that will document user identity and timestamps for all entries, modifications, and deletions.

### Optimizing retention and follow-up adherence

Participants will receive comprehensive information regarding trial procedures and expectations during the study period, with particular emphasis on the critical importance of follow-up compliance. A structured 3-month follow-up protocol will be provided to all patients,who will be strongly encouraged to attend scheduled clinic visits for face-to-face assessments. Two days prior to each follow-up appointment, designated research staff will proactively contact participants via telephone, WeChat, or alternative preferred communication channels to confirm attendance. For individuals unable to complete in-person evaluations, remote cognitive assessments will be facilitated through validated software platforms [https://doctor.shenzhenheling.com/],enabling flexible self-administration at the participant’s convenience. All remotely collected data will undergo immediate verification by research personnel, with subsequent outreach to be conducted as necessary to resolve missing or inconsistent responses. Participants retain the right to withdraw from follow-up procedures at any time without consequence to their standard medical care.

### Statistical methods

#### Clinical data analysis.

Statistical analyses will be conducted using SPSS 26.0, with a two-tailed significance level of p < 0.05. Categorical variables will be presented as frequencies and percentages, and compared using chi-square tests. Continuous variables will be assessed for normality using the Shapiro-Wilk test; normally distributed data will be reported as mean±SD, and non-normally distributed data as median [IQR].For repeated measures (e.g.,cognitive assessments at multiple timepoints), linear mixed models (LMM) will be used to include covariates (e.g., age, baseline scores, education level) and manage missing data. Between-group comparisons at individual timepoints will be conducted using one-way ANOVA for normally distributed data or Kruskal-Wallis tests for non-normal data, with multiple comparisons adjusted using the false discovery rate (FDR) method. Correlations will be analyzed using Pearson’s correlation for normally distributed data and Spearman’s correlation for non-normal data.The primary outcome analysis will be conducted on an intention-to-treat (ITT) basis [26]. Missing data will be addressed using complete case analysis if the missing rate is less than 5%, or multiple imputation if the missing rate is 5% or higher [27].

#### FNIRS data analysis.

The fNIRS data will be systematically analyzed using the NirSpark software package (v1.7.5, Danyang Huichuang Medical Equipment Co., Ltd., China). The preprocessing pipeline will include motion artifact correction based on moving standard deviation (Moving SD) detection combined with cubic spline interpolation to eliminate signal jumps caused by head movements. Subsequently, a bandpass filter with a cutoff frequency of 0.02–0.20 Hz will be applied to remove physiological noise such as heartbeats and respiration. The preprocessed optical density signals will be then converted into relative changes in HbO and HbR)concentrations using the modified Beer-Lambert law. HbO will be selected as the primary analytical indicator due to its higher signal-to-noise ratio. For the VFT phase, the average HbO concentration will be calculated over a 60-second time window following task onset, with baseline drift will be corrected using linear fitting. To extract group-level hemodynamic features, the individual HbO signals across all 19 channels from both experimental groups will be averaged at the group level. Finally, HbO concentration topographic maps and functional connectivity maps of the right prefrontal cortex (channels 1–11) and left prefrontal cortex (channels 12–19) will be generated using the NirSpark software to reflect inter-regional brain interactions.

### Dissemination

The dissemination of scientific findings will be achieved through publication in international peer-reviewed journals. This includes the transparent reporting of all study outcomes, encompassing both positive and negative results.

## Discussion

PSCI remains a significant and unmet challenge in rehabilitation, greatly affecting functional recovery and quality of life. This randomized controlled trial will rigorously evaluate the clinical efficacy of sequential tDCS-CCT interventions for PSCI. Based on previous literature, the sequence of combined interventions may lead to different therapeutic outcomes through distinct neuroplastic mechanisms, as the effects of tDCS are time-dependent, and the impact of stimulation timing exhibits complexity and bidirectionality [28–30]. When the training task is synchronized with the tDCS-induced state of neural excitation, tDCS can optimize neuronal firing synchrony, thereby enhancing synaptic efficiency and cognitive processing through activity-dependent synaptic plasticity. If tDCS is administered prior to CCT, it may initiate neuroplastic networks through a synapse remodeling process similar to LTP; however, the potential interference from homeostatic plasticity mechanisms must be considered. According to the Bienenstock–Cooper–Munro (BCM) theory, the enhancement of postsynaptic activity induced by tDCS increases the modification threshold (MT), thereby reducing the likelihood of LTP and increasing the probability of long-term depression (LTD), which may ultimately impair learning performance [31].In contrast, administering tDCS after training may stabilize newly formed neural connections through activity-dependent synchronization. We hypothesize that the synchronous strategy, by maximizing the immediate induction of LTP, yields the most optimal intervention outcome.

The brain’s high metabolic demand—accounting for 20% of total oxygen consumption despite representing only 2% of body weight—necessitates close monitoring of cerebral hemodynamics [32,33]. Chronic hypoperfusion triggers pathological cascades, including microvascular dysfunction, neuronal injury, and cortical atrophy, ultimately disrupting the functional networks essential for cognition [34]. Functional neuroimaging confirms task-induced neurovascular coupling, wherein activated neurons drive local arteriolar dilation [35,36]. Chen et al.’s tDCS-CCT study demonstrated enhanced cerebrovascular reactivity and regional blood flow, highlighting the utility of HbO as a sensitive biomarker for microvascular changes that predict cognitive recovery.

Methodological limitations must be considered: neuropsychological testing is susceptible to ceiling and practice effects in longitudinal designs. High-functioning patients may experience premature plateaus (“ceiling effect”), while low-functioning individuals may show artificial improvements due to repeated exposure (“practice effect”), complicating the assessment of true intervention effects.Notably, inter-individual variance in lesion site and severity will modulate treatment response; we will therefore integrate baseline imaging—lesion location and volume—for subgroup analyses to craft interventions that concurrently respect lesion signature, disease stage and temporal order. As evidence on the tDCS–CCT pairing will remain sparse and prior cohorts will have been small, heterogeneity will persist; thus, the forthcoming findings will generalize only to the single-center Chinese cohort to be recruited here, and subsequent larger, geographically dispersed trials will be required to validate this sequential strategy.

### Composition of the data monitoring committee, its role, and reporting structure

In accordance with recommendations from the Data Safety Monitoring Board (DSMB) of Shenzhen Nanshan People’s Hospital, a dedicated DSMB has not been appointed for this study. As all intervention protocols in this trial constitute standard clinical practices within our department, DSMB interim assessments would not confer additional safety benefits. In the event of a serious adverse event (SAE), the department director will be notified within 48 hours to evaluate whether the SAE is definitively or possibly treatment-related. Should a plausible causal relationship be identified, subsequent safety measures will be implemented following consultation with the designated safety officer, in accordance with established risk management protocols.

### Frequency and plans for auditing trial conduct

The research group will convene on a monthly basis to review the progress of the trial and address any potential issues that may arise. Furthermore, an independent monitor will annually verify the presence and integrity of the trail documentation, which include informed consent, inclusion and exclusion criteria, as well as source data collection.

## Supporting information

S1 File SPIRIT checklist. The completed Standard Protocol Items: Recommendations for Interventional Trials (SPIRIT) 2013 checklist for this study protocol.
(PDF)

## Abbreviations

PSCI: Post-stroke cognitive impairment
tDCS: transcranial direct current stimulation
CCT: computerized cognitive training
fNIRS: functional near-infrared spectroscopy
MoCA: Montreal Cognitive Assessment
MMSE: Mini-Mental State Examination
BI: Barthel Index
HAMA: Hamilton Anxiety Rating Scale
HAMD: Hamilton Depression Rating Scale
DLPFC: dorsolateral prefrontal cortex
PFC: Prefrontal Cortex
HbO: oxygenated hemoglobin
VFT: Verbal Fluency Task
